# Prognostic Value of Preoperative Systemic Immune-Inflammation Index in Patients with Cervical Cancer

**DOI:** 10.1038/s41598-019-39150-0

**Published:** 2019-03-01

**Authors:** Huaping Huang, Qin Liu, Lixia Zhu, Yan Zhang, Xiaojuan Lu, Yawei Wu, Li Liu

**Affiliations:** 1 Department of obstetrics, The First People’s Hospital of Kunshan, Jiangsu University, No. 91 West Qianjin Road, Suzhou, 215300 China; 2 Department of Gynecology, The First People’s Hospital of Kunshan, Jiangsu University, No. 91 West Qianjin Road, Suzhou, 215300 China

## Abstract

The systemic immune-inflammation index (SII) based on peripheral lymphocyte, neutrophil and platelet counts has been considered a good index that reflects the local immune response and systemic inflammation. However, the use of the SII has not been reported in cervical cancer. In this study, Kaplan-Meier survival analysis showed that a high SII was associated with poor prognosis in cervical cancer patients in the primary and validation cohorts. A higher SII had a significant correlation with larger tumours but had no correlation with other clinicopathological parameters. Among all systemic immune indexes, the SII is the only independent prognostic factor for cervical cancer patients. Compared with the area under the curve for the neutrophil/lymphocyte ratio (NLR), platelet/lymphocyte ratio (PLR), and monocyte/lymphocyte ratio (MLR), the area for the SII was larger at 3 and 5 years. In addition, the SII still retains it prognostic values across all FIGO stages. The SII can independently predict the overall survival of patients with cervical cancer receiving radical resection and is thus superior to existing systemic inflammatory indexes. The prognostic nomogram based on the SII is a reliable model for predicting the postoperative survival of patients with cervical cancer.

## Introduction

Cervical cancer is a common malignant tumour in females, and its incidence rate is second only to breast cancer, seriously threatening female health and life. Developing countries have a high prevalence of cervical cancer, with their incidence accounting for 90% of the total^1^. Currently, radical hysterectomy is the dominant treatment for early cervical cancer. With the popularization of cervical cancer screening, the efficacy of treatment and the prognosis for the increasing number of early-stage patients have been greatly improved. Cervical cancer in the middle-advanced stage is mainly treated with radiotherapy and/or chemotherapy, but the therapeutic effect is still unsatisfactory^2,3^. Postoperative recurrence and metastasis of cervical cancer are the main causes of death in the clinic. At present, the International Federation of Gynaecology and Obstetrics (FIGO) staging system is the main evaluation criterion used by clinicians to predict the prognosis of patients with cervical cancer. However, the prognoses differ among cervical cancer patients in similar clinical stages. Therefore, the hierarchical processing of high-risk subgroups is rational and necessary for individualized monitoring and optimized postoperative treatment.

In recent years, the tumour microenvironment has received increasing attention^4^, and a variety of inflammatory cells and inflammatory mediators are important components of the tumour microenvironment. The peripheral leukocytes, neutrophils, lymphocytes, platelets and acute-phase proteins contribute to the inflammatory response and can be detected in an easy and convenient way. In recent years, a number of studies have demonstrated that the systemic inflammatory response is related to the postoperative survival of tumour patients^5–11^. The indexes with prognostic value include the tumour-related leucocytosis (TRL), neutrophil/lymphocyte ratio (NLR), platelet/lymphocyte ratio (PLR) and monocyte/lymphocyte ratio (MLR). Recently, the SII based on peripheral lymphocyte, neutrophil and platelet counts has been considered as a better index to reflect the local immune response and systemic inflammation, as its high prognostic value has been confirmed in a variety of tumours, such as hepatocellular cancer^12^, oesophageal cancer^13,14^, colorectal cancer^15^ and small cell lung cancer^16^. However, the correlation between the SII and postoperative survival has not been evaluated in cervical cancer patients. In this study, the value of systemic inflammatory indexes, including the SII, NLR, PLR and MLR, in predicting the prognosis of patients with cervical cancer was evaluated in two independent cohorts.

## Results

### Patients’ characteristics

A total of 458 patients with cervical cancer were enrolled in this study. The clinicopathological features of patients in the primary cohort and validation cohort are shown in Table 1. In the primary cohort, the median follow-up time was 47 months (3–129 months), the median age of patients was 45 years old (22–86 years old), and the average number of lymph nodes obtained during surgical resection was 24.8 (10–74). In the validation cohort, the median follow-up time was 47 months (3–120 months), and the median age of patients was 44 years old (23–68 years old). Patients in the two cohorts had similar clinicopathological features except histological grade. The validation cohort has more patients with grade II.

**Table 1:** Clinicopathological characteristics of patients with cervical cancer in primary cohort and validation cohort.

Characteristic	Primary Cohort (n = 328)		Validation Cohort (n = 130)		χ^2^	*P*
	No. of Patients	%	No. of Patients	%		
Age					0.29	0.590
≤45	170	51.8	71	54.6		
>45	158	48.2	59	45.4		
Histological grade					15.66	0.001
G1	36	11.0	12	9.2		
G2	141	43.0	82	63.1		
G3	151	46.0	36	27.7		
Tumor size					0.99	0.319
≤4	180	54.9	78	60.0		
>4	148	45.1	52	40.0		
Lymph node metastasis					1.07	0.301
Negative	257	78.4	96	73.8		
Positive	71	21.6	34	26.2		
Lymphovascular invasion					1.78	0.182
No	288	87.8	108	83.1		
Yes	40	12.2	22	16.9		
FIGO stage					0.02	0.99
IA	84	25.6	34	26.2		
IB	174	53.0	68	52.3		
IIA	70	21.3	28	21.5		

### Correlation between SII and clinicopathological variables

Based on the definition of the cut-off value, the SII group was divided into a high-SII group and a low-SII group. In the primary cohort, there were 170 (51.8%) patients in the low-SII group and 158 (48.2%) patients in the high-SII group. It was found that a higher SII had a significant correlation with larger tumours (*P* < 0.001) but had no correlation with other clinicopathological parameters (Table 2). In addition, the SII was significantly correlated with other systemic immune markers (NLR, PLR and MLR). In the validation cohort, there were 47 (36.2%) patients in the low-SII group and 83 (63.8%) patients in the high-SII group. The results for this cohort also confirmed that higher SII had a significant correlation with large tumours but had no correlation with other clinicopathological parameters (Table 2).

**Table 2:** Correlations between preoperative SII and clinicopathological characteristics in primary and validation cohort.

Clinical parameter	Primary Cohort				Validation Cohort			
	SII ≤ 475 (170)	SII > 475 (158)	χ^2^	*P*	SII ≤ 475 (47)	SII > 475 (83)	χ^2^	*P*
Age			0.22	0.641			2.93	0.087
≤45	86	84			21	50		
>45	84	74			26	33		
Histological grade			3.08	0.215			4.19	0.123
G1	22	14			5	7		
G2	77	64			34	48		
G3	71	80			8	28		
Tumor size			32.59	<0.001			30.41	<0.001
≤4	119	61			43	35		
>4	51	97			4	48		
Lymph node metastasis			0.10	0.747			0.91	0.341
Negative	132	125			37	59		
Positive	38	33			10	24		
Lymphovascular invasion			1.22	0.270			0.01	0.982
No	146	142			39	69		
Yes	24	16			8	14		
FIGO stage			1.62	0.445			2.37	0.305
IA	42	42			16	18		
IB	87	87			22	46		
IIA	41	29			9	19		
TRL			37.77	<0.001			8.40	0.004
TRL (−)	163	112			45	63		
TRL (+)	7	46			2	20		
NLR			83.69	<0.001			52.19	<0.001
NLR ≤ 2.4	132	43			44	23		
NLR>2.4	38	115			3	60		
PLR			59.55	<0.001			26.55	<0.001
PLR ≤ 118	106	32			35	23		
PLR > 118	64	126			12	60		
MLR			16.42	<0.001			10.70	0.001
MLR ≤ 0.26	124	81			26	22		
MLR>0.26	46	77			21	61		

SII: systemic immune-inflammation index; TRL: tumour-related leucocytosis; NLR: neutrophil lymphocyte ratio; PLR: platelet lymphocyte ratio; MLR: monocyte lymphocyte ratio.

### Correlation between systemic inflammatory indexes and the prognosis of patients with cervical cancer

**Figure 1:**
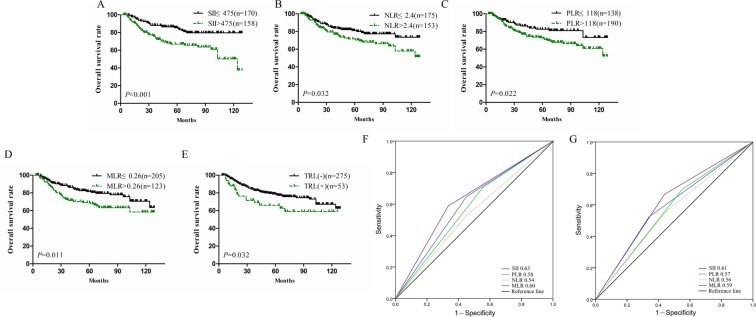
The prognostic significance of the SII (**A**), NLR (**B**), PLR (**C**), MLR (**D**) and TRL (**E**) in cervical cancer in the primary cohort. Predictive ability of the SII in cervical cancer was compared with PLR, NLR and MLR by ROC curves in 3-years (**F**) and 5-years (**G**) in the primary cohort.

**Figure 2:**
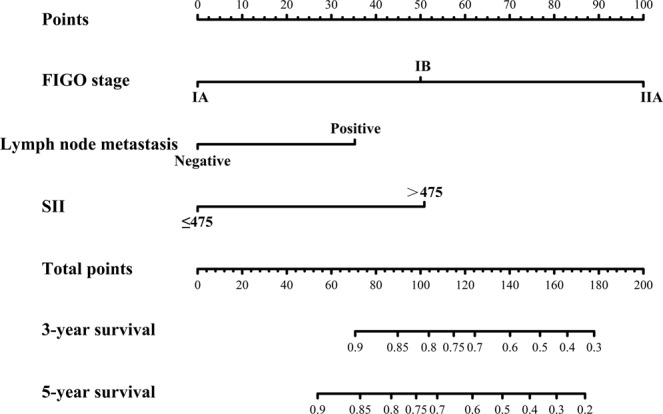
Evaluation of nomogram integrated SII, FIGO stage and Lymph node metastasis in patients with cervical cancer. The risk factors in nomogram consisted of FIGO stage, lymph node metastasis and SII. According to the above three parameters of a patient, the corresponding values were found in each variable axis. Then, at each point of the three values, a vertical line was made upward to intersect with the upper ruler, and the intersection represented a score. Calculate the total score by adding the three scores, and find it on the lower ruler. Make a vertical line down to intersect with the 3-year survival rate and the 5-year survival rate lines. Finally, the predicted 3-year survival rate and 5-year survival rate of this patient were determined.

**Figure 3:**
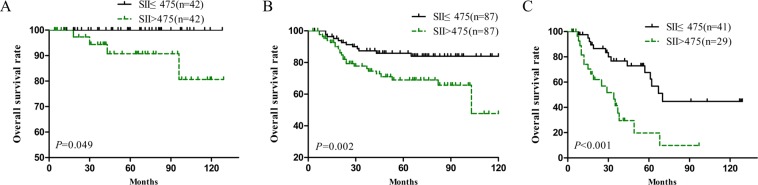
The prognostic significance of the SII based on FIGO IA (**A**), FIGO IB (**B**), and FIGO IIA (**C**) in cervical cancer in the primary cohort.

The results of survival analysis for patients in the primary cohort stratified by SII, NLR, PLR and MLR are shown in Fig. 1. As shown in Fig. 1A, the survival time in the high-SII group was significantly shorter than that in the low-SII group, indicating that the prognosis is poorer in the high-SII group. Similarly, the higher the NLR, PLR, and MLR were, the shorter the survival time was (Fig. 1B–D). OS was significantly shorter in TRL (+) patients than TRL (−) patients (Fig. 1E). Then, univariate Cox regression analysis was performed for the clinicopathological parameters and systemic immune indexes included in Table 2, and the results showed that the tumour size, lymph node metastasis, lymphovascular invasion, FIGO stage, SII, TRL, NLR, PLR and MLR had a statistically significant effect on the survival time of patients with cervical cancer. A tumour size >4, positive lymph node metastasis, lymphovascular invasion, advanced FIGO stage, high SII, high NLR, TRL (+), high PLR and high MLR were risk factors for poor prognosis in patients with cervical cancer (Table 3). After baseline adjustment of univariate indexes, multivariate analysis was performed, and the results revealed that the SII, lymph node metastasis and FIGO stage were independent prognostic factors for cervical cancer patients. We also built a nomogram based on all variables that are significant in the multivariate analysis. The FIGO stage, lymph node metastasis and SII were eventually integrated in the nomogram to predict the 3- and 5-year survival of cervical cancer patients (Fig. 2). According to the above three parameters of a patient, the corresponding values were found in each variable axis. Then, at each point of the three values, a vertical line was made upward to intersect with the upper ruler, and the intersection represented a score. Calculate the total score by adding the three scores, and find it on the lower ruler. Make a vertical line down to intersect with the 3-year survival rate and the 5-year survival rate lines. Finally, the predicted 3-year survival rate and 5-year survival rate of this patient were determined (Supplemental Fig. 1). Among all systemic immune indexes, the SII is the only independent prognostic factor for cervical cancer patients. Then, the ROC curve was used to judge the ability of systemic immune indexes to predict prognosis. Compared with the area under the curve for NLR, PLR and MLR, the area was larger for the SII at 3 and 5 years (Fig. 1F,G). It can be concluded that the SII is able to distinguish the prognosis of patients with cervical cancer. In conclusion, it is believed that the SII is significantly superior to other systemic immune indexes. In addition, the ability of the SII to distinguish the prognosis of patients in different FIGO stages was verified. In FIGO stages IA, IB and IIA, the prognosis of cervical cancer patients with a high SII was poorer than that of low-SII patients in the same stages (Fig. 3).

**Table 3:** Univariate and multivariate cox regression analyses for overall survival in cervical cancer patients in primary cohort.

Variables	Univariate analysis		Multivariate analysis	
	HR (95%CI)	*P* value	HR (95%CI)	*P* value
Age				
≤45 years vs. >45years	0.98 (0.62–1.54)	0.929		
Histological grade		0.031		0.681
G1	Ref.	—	Ref.	
G2	1.46 (0.51–4.18)	0.486	0.76 (0.26–2.21)	0.608
G3	2.53 (0.91–7.03)	0.075	0.93 (0.32–2.70)	0.899
Tumor size				
>4 vs. ≤4	2.35 (1.46–3.78)	<0.001	1.21 (0.72–2.04)	0.475
Lymph node metastasis				
Positive vs. Negative	4.77 (2.83–8.04)	<0.001	2.74 (1.51–4.98)	0.001
Lymphovascular invasion				
Yes vs. No	2.53 (1.02–6.28)	0.045	1.84 (0.69–4.96)	0.221
FIGO stage		<0.001		<0.001
IA	Ref.		Ref.	
IB	4.97 (1.77–13.90)	0.002	3.06 (1.03–9.62)	0.048
IIA	14.00 (4.94–39.68)	<0.001	7.33 (2.24–24.05)	0.001
SII				
>475 vs. ≤475	2.46 (1.52–3.96)	<0.001	2.53 (1.32–4.83)	0.005
TRL				
TRL (+) vs. TRL (−)	1.79 (1.04–3.07)	0.035	1.14 (0.60–2.14)	0.691
NLR				
>2.4 vs. ≤2.4	1.64 (1.04–2.60)	0.020	1.24 (0.73–2.09)	0.423
PLR				
>118 vs. ≤118	1.77 (1.08–2.91)	0.025	1.38 (0.75–2.53)	0.297
MLR				
>0.26 vs. ≤0.26	1.79 (1.14–2.81)	0.012	1.14 (0.69–1.93)	0.653

SII: systemic immune-inflammation index; TRL: tumour-related leucocytosis; NLR: neutrophil lymphocyte ratio; PLR: platelet lymphocyte ratio; MLR: monocyte lymphocyte ratio; HR: hazard ratio; CI: confidence interval; Ref: reference.

### Verification of the prognostic value of the SII in cervical cancer

**Figure 4:**
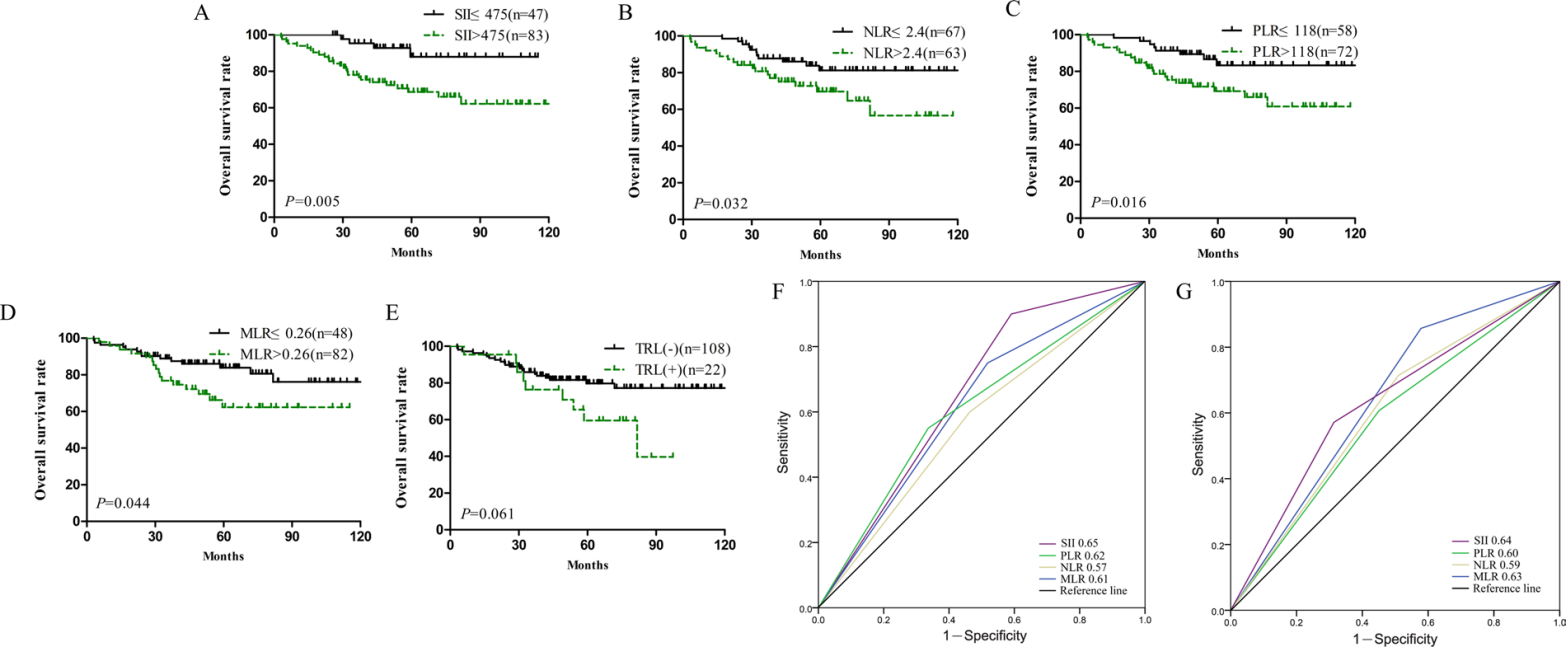
The prognostic significance of the SII (**A**), NLR (**B**), PLR (**C**), MLR (**D**) and TRL (**E**) in cervical cancer in the validation cohort. Predictive ability of the SII in cervical cancer was compared with PLR, NLR and MLR by ROC curves in 3-years (**F**) and 5-years (**G**) in the validation cohort.

The above results were verified in a separate cohort. The survival curve showed that the survival time of patients with high SII, high NLR, high PLR and high MLR values was significantly shorter than that of patients with low values (Fig. 4A–D). TRL (+) patients maybe have shorter survival time than TRL (−) patients was a, but no statistical difference (Fig. 4E). The univariate analysis showed that the tumour size, lymph node metastasis, FIGO stage, SII, NLR, PLR and MLR had a statistically significant effect on the survival time of patients with cervical cancer (Table 4). Multivariate analysis revealed that the SII, lymph node metastasis and FIGO stage were independent prognostic factors for cervical cancer patients (Table 4). According to the ROC curve analysis, the SII had a larger area under the curve at 3 and 5 years compared with the area found for NLR, PLR and MLR (Fig. 4F,G). The prognostic value of the SII in cervical cancer was confirmed through the independent validation cohort.

**Table 4:** Univariate and multivariate cox regression analyses for overall survival in cervical cancer patients in validation cohort.

Variables	Univariate analysis		Multivariate analysis	
	HR (95%CI)	*P* value	HR (95%CI)	*P* value
Age				
≤45 years vs. >45years	0.70 (0.34–1.48)	0.354		
Histological grade		0.454		
G1	Ref.	—		
G2	3.40 (0.46–25.29)	0.232		
G3	2.74 (0.34–21.94)	0341		
Tumor size				
>4 vs. ≤4	2.57 (1.24–5.35)	0.011	1.87 (0.80–4.40)	0.150
Lymph node metastasis				
Positive vs. Negative	3.12 (1.52–6.41)	0.002	2.35 (1.05–4.55)	0.043
Lymphovascular invasion				
Yes vs. No	1.86 (0.83–4.19)	0.134		
FIGO stage		0.002		0.001
IA	Ref.		Ref.	
IB	2.50 (0.72–8.71)	0.151	2.12 (0.61–7.40)	0.240
IIA	6.92 (1.97–24.33)	0.003	6.60 (1.88–23.22)	0.003
SII				
>475 vs. ≤475	4.04 (1.41–11.59)	0.009	3.99 (1.38–11.47)	0.010
TRL				
TRL (+) vs. TRL (−)	2.08 (0.95–4.54)	0.067		
NLR				
>2.4 vs. ≤2.4	2.21 (1.05–4.65)	0.037	1.96 (0.91–4.20)	0.083
PLR				
>118 vs. ≤118	2.62 (1.16–5.88)	0.020	2.33 (0.92–5.90)	0.075
MLR				
>0.26 vs. ≤0.26	2.06 (1.00–4.21)	0.049	1.73 (0.83–3.60)	0.141

SII: systemic immune-inflammation index; TRL: tumour-related leucocytosis; NLR: neutrophil lymphocyte ratio; PLR: platelet lymphocyte ratio; MLR: monocyte lymphocyte ratio; HR: hazard ratio; CI: confidence interval; Ref: reference.

## Discussion

The association between inflammation and cancers was first noticed by Virchow in 1863^17^. Inflammation is a kind of anti-injury response to endogenous or exogenous injury in the body, and tumour-associated inflammation plays an important role in the development of tumours^18^. In the early stage of tumour development, inflammation has the potential to promote cell mutagenesis^19^. The chronic inflammation microenvironment included a lot of inflammatory cells especially macrophages^20^. These inflammatory cells generate high levels of reactive oxygen (ROS), nitrogen, tumour necrosis factor-α and macrophage migration inhibitory factor^19^. The persistence of these infection-fighting agents caused mutations by exacerbating DNA damage in proliferating cells^19^. During the progression of tumour development, tumour cells transform some inflammatory substrates into mediators that support tumour spread and metastasis^19^. Tumour cells can produce cytokines and chemokines to attract immune cells to facilitate cancer development^21–23^. For example, tumour-associated macrophages (TAM) release interleukin (IL)-10 and prostaglandin E_2_ to suppress antitumor response^24^. TAMs may also facilitate tumor cell invasion and metastasis by releasing MMP-2 and MMP-9, which degrade the extracellular matrix and the basement membrane^25^. Moreover, TAMs release epidermal growth factor and other epidermal growth factor receptor family ligands to promote tumour cell proliferation and migration^26^. In previous studies, the systemic inflammatory response has been proved to be a factor in the poor prognosis of patients with various cancers. To investigate the simple and effective prognostic indexes used in the evaluation of the prognosis and the guidance of clinical treatment of cervical cancer patients, many indexes based on inflammation were studied and discussed in this paper, and this exploration focused on the value of the SII in the prognostic evaluation of cervical cancer patients.

NLR is a marker for the general immune response to various stress stimuli^27^. Under inflammatory conditions, neutrophil precursors, such as myelocytes and promyelocytes, may be released^28^. The increased neutrophil population can secrete a large amount of nitric oxide, arginase and ROS, resulting in disorders of T cell activation^29^ and production of vascular endothelial growth factor (VEGF), leading to tumour neovascularization^30^. The measurement of circulating neutrophils alone or as a part of NLR can predict the outcomes of patients in various cancers. Platelets are part of the inflammatory response, and there are often increased numbers of platelets in patients with solid tumours^31^. Currently, it is believed that thrombopoietin is secreted by tumour cells, just as interleukin-6 is. Platelets can directly act on tumour cells to benefit tumour growth, invasion and angiogenesis^32^. Lymphocytes can secrete cytokines such as IFN-γ and TNF-α, thus controlling tumour growth and improving the prognosis of cancer patients^33^. The decline in the number and function of lymphocytes can weaken immunological surveillance and defence^20,33^. Therefore, a decline in lymphocytes predicts cellular immune injury, and increased numbers of neutrophils and platelets are considered responses to systemic inflammation^34^. Therefore, the SII based on the above three kinds of inflammatory cells can better display the balance between pro-tumour and anti-tumour immune status in cancer patients.

In this study, the ability of the SII, TRL, NLR, PLR and MLR to predict postoperative survival of patients with cervical cancer was studied. It was confirmed that the SII, TRL, NLR, PLR and MLR could be used to judge the prognosis of patients after radical surgery for cervical cancer treatment. According to the multivariate analysis, only the SII was an independent prognostic factor for patients with cervical cancer. The ROC curve analysis showed that, among these markers, the SII was more effective and accurate in predicting the outcomes of patients. Before our study, there are multiple papers that look at TRL and outcomes in cervical cancer^9–11,35,36^. TRL was indeed a risk factor for patients with cervical cancer. However, TRL has an obvious defect compared with SII. When classified by the TRL, there were 85% of patients classified in the group of TRL (−), which meant that the TRL couldn’t distinguish the survival differences of most of the patients. Therefore, it is believed that the SII is a more objective marker of the balance between host inflammation and immune response status. In addition, the above results were validated in an independent validation cohort, adding to the reliability of the results.

There were also some limitations to this study. First, selection bias may exist in this study, as it is a retrospective study, or there may be detection bias and analysis bias. Second, SII, MLR and TRL had a difference between the primary cohort and validation cohort in our study. The main reason is that the cut-off value is generated from the primary cohort, and the validation cohort applies this cut-off value. However, the best cut-off value from the primary cohort is not necessarily the most suitable for validation cohort, and therefore leads to selection bias. The second reason is that the two central laboratories where the two cohorts are located use different detection reagents and instruments, so there are some differences in the test data. The third possible reason is that peripheral blood cell analysis results are easily affected by factors such as blood circulation capacity, infection, and nutritional status. It is reasonable to have different blood cell analysis results, SII, MLR and TRL between the two cohorts. Since the best cut-off value should be obtained using the ROC curve in a single cohort. The solution to this problem is to conduct a single-center, large-sample study. Moreover, the treatment of patients after surgical resection has some heterogeneity, leading to different clinical outcomes.

In conclusion, the systemic inflammatory response of patients with cervical cancer can predict the postoperative survival outcome. In particular, a high SII is closely related to poor prognosis in cervical cancer patients. The SII can distinguish the prognosis of patients in different FIGO stages, which is an important supplement to FIGO stage. High-risk patients can be screened more sensitively and the SII can be specifically combined with the FIGO stage to determine the optimal individualized treatment. In conclusion, the SII is a universally available method characterized by a non-invasive approach, easy access and low cost, and it thus has promising prospects for application.

## Methods

### Patients

A total of 328 patients with cervical cancer receiving radical resection in the Second Affiliated Hospital of Soochow University from 2006 to 2015 were recruited as the primary cohort. Another 130 patients with cervical cancer receiving radical resection in the First People’s Hospital of Kunshan were enrolled as the validation cohort. The pathological type of all patients was cervical squamous cell carcinoma, and routine blood examination was performed before the operation. The study excluded patients who had other pathological types; who underwent preoperative neoadjuvant radiotherapy and/or chemotherapy; who had distant metastasis in the liver, lung, or peritoneum/pelvic cavity diagnosed before or during the operation; or who had active infection or inflammatory diseases within the month before blood examination. The FIGO staging criteria were used for the tumour staging. This retrospective study was approved by the Ethics Committee of the First People’s Hospital of Kunshan. This study was undertaken according to the Declaration of Helsinki. Written informed consent was obtained from all participants.

### Data collection

Clinical data were collected from patients, including the clinicopathological parameters (such as age, histologic grade, tumour size, lymph node metastasis, lymphovascular space invasion, adjuvant therapy and FIGO stages) and preoperative routine blood examination results (absolute counts of leukocytes, neutrophils, lymphocytes and platelets).

Venous blood was drawn from all patients within the week before surgery. The SII was calculated using the following equation: SII = platelet count × neutrophil count/lymphocyte count. The cut-off value was determined as follows: The receiver operating characteristic (ROC) curve was plotted to determine the cut-off value of the haematological index and tumour size for predicting the survival rate of patients. The upper right point of the ROC curve was selected, the Youden index was calculated according to the sensitivity and specificity of each possible point in the statistical results, and the largest point of contact was selected as the cut-off to determine the optimal cut-off value of the haematological index and tumour size in the ROC curve, so that the numerical variable could be transformed into a classified variable for the analysis of statistical data^37^. According to the above method, the optimal cut-off values obtained are as follows: SII (SII ≤ 475, SII > 475), NLR (NLR ≤ 2.4, NLR > 2.4), PLR (PLR ≤ 118, PLR > 118) and MLR (MLR ≤ 0.26, MLR > 0.26). The cut-off value of TRL was based on previous literature and clinical practice. TRL (+) was defined as the absolute leukocyte count exceeding 9000/µL.

### Follow-up

After the initial treatment, follow-up intervals were once every 3 months for years 1 and 2, once every 6 months for years 3–5, and then once every year after year 5. The main examinations included routine blood tests, hepatic and renal function tests, tumour marker measurements, vaginal examination, chest radiography, and CT or MRI scans. In cases with abnormal findings and suspected tumour recurrence, lesion biopsy was performed to determine whether recurrence occurred.

### Statistical analysis

The chi-square test or Fisher exact probability method was used for the comparison of classified variables between two groups. A t-test or Mann-Whitney U test was used for the analysis of differences in the numerical variables between two groups. The area under the curve of index was calculated using the ROC curve to compare the accuracy of each systemic inflammatory index in predicting patient outcomes. The Cox proportional hazard model was used to perform univariate and multivariate analyses of the factors influencing the prognosis of patients. The survival curve was plotted using the Kaplan-Meier method to calculate the survival rate, and the log-rank test was performed to compare the survival rates between the two groups. SPSS 20.0 and GraphPad Prism 5 statistical software were used for the data analysis. All *P* values were bilaterally distributed, and *P* < 0.05 indicated that the difference was statistically significant.

## Supplementary information

Supplemental fugure 1

## Data Availability

The data used to support the findings of this study are available from the corresponding author upon request.
