# Supplementary material for: Prognostic Value of Preoperative Systemic Immune-Inflammation Index in Patients with Cervical Cancer
Source: Sci Rep. 2019 Mar 1;9:3284. doi: 10.1038/s41598-019-39150-0 (PMC6397230; doi:10.1038/s41598-019-39150-0)
Supplement: Supplementary file 1 — Supplemental fugure 1 [file 41598_2019_39150_MOESM1_ESM.pdf]

Prognostic Value of Preoperative Systemic Immune-Inflammation Index in Patients with Cervical Cancer

Huaping Huang[1], Qin Liu[2], Lixia Zhu[2], Yan Zhang[2], Xiaojuan Lu[2], Yawei Wu[2], Li Liu[1,]

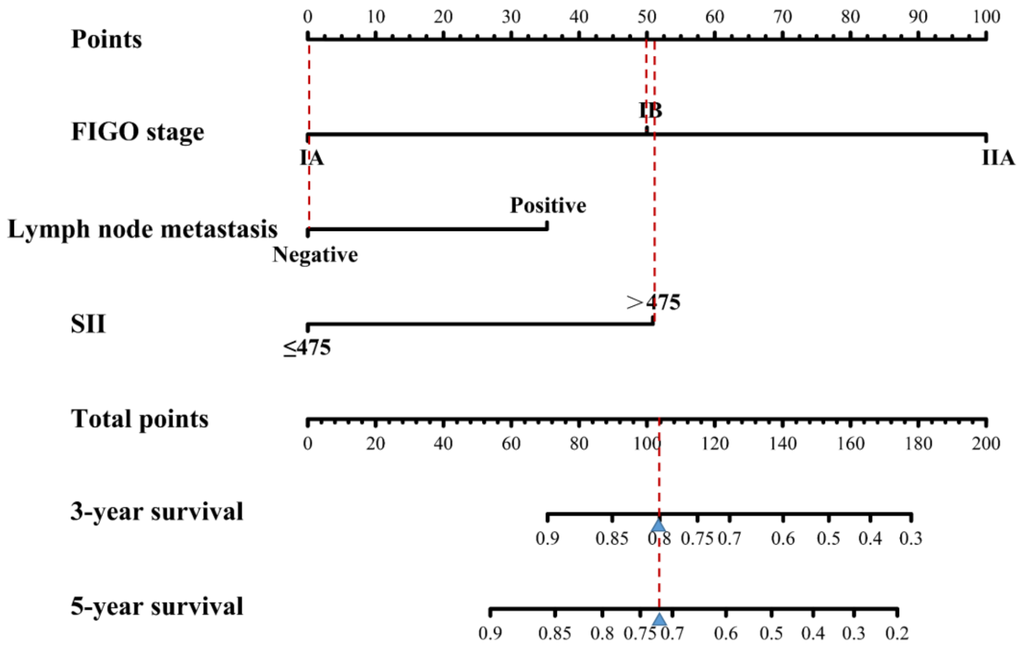

If there is a cancer patient with FIGO stage IB, lymph node metastasis negative and a SII> 475, first, find the three points on the horizontal lines of the three variables, and then make a vertical line from the three points. According to the ruler above, the scores of these three variables are 50 points, 0 points and 52 points respectively. Add up the three scores and get a total score of 102 points. Find the 102 points on the lower ruler and make a vertical line down to intersect with the 3-year survival rate and the 5-year survival rate lines. Finally, the predicted 3-year survival rate and 5-year survival rate of this patient are 80% and 72%, respectively
